# Association between DNA Damage Response and Repair Genes and Risk of Invasive Serous Ovarian Cancer

**DOI:** 10.1371/journal.pone.0010061

**Published:** 2010-04-08

**Authors:** Joellen M. Schildkraut, Edwin S. Iversen, Melanie A. Wilson, Merlise A. Clyde, Patricia G. Moorman, Rachel T. Palmieri, Regina Whitaker, Rex C. Bentley, Jeffrey R. Marks, Andrew Berchuck

**Affiliations:** 1 Departments of Community and Family Medicine, Duke University Medical Center, Durham, North Carolina, United States of America; 2 The Duke Comprehensive Cancer Center, Duke University Medical Center, Durham, North Carolina, United States of America; 3 Department of Statistical Science, Duke University Medical Center, Durham, North Carolina, United States of America; 4 Department of Epidemiology, Gillings School of Global Public Health, University of North Carolina, Chapel Hill, North Carolina, United States of America; 5 Obstetrics and Gynecology/Division of Gynecologic Oncology, Duke University Medical Center, Durham, North Carolina, United States of America; 6 Department of Pathology, Duke University Medical Center, Durham, North Carolina, United States of America; 7 Department of Surgery, Duke University Medical Center, Durham, North Carolina, United States of America; 8 Institute for Genome Sciences and Policy, Duke University Medical Center, Durham, North Carolina, United States of America; Universite de Montreal, Canada

## Abstract

**Background:**

We analyzed the association between 53 genes related to DNA repair and p53-mediated damage response and serous ovarian cancer risk using case-control data from the North Carolina Ovarian Cancer Study (NCOCS), a population-based, case-control study.

**Methods/Principal Findings:**

The analysis was restricted to 364 invasive serous ovarian cancer cases and 761 controls of white, non-Hispanic race. Statistical analysis was two staged: a screen using marginal Bayes factors (BFs) for 484 SNPs and a modeling stage in which we calculated multivariate adjusted posterior probabilities of association for 77 SNPs that passed the screen. These probabilities were conditional on subject age at diagnosis/interview, batch, a DNA quality metric and genotypes of other SNPs and allowed for uncertainty in the genetic parameterizations of the SNPs and number of associated SNPs. Six SNPs had Bayes factors greater than 10 in favor of an association with invasive serous ovarian cancer. These included rs5762746 (median OR(odds ratio)_per allele_ = 0.66; 95% credible interval (CI) = 0.44–1.00) and rs6005835 (*median* OR_per allele_ *= 0.69; 95% CI  = 0.53–0.91*) in *CHEK2*, rs2078486 (median OR_per allele_  = 1.65; 95% CI = 1.21–2.25) and rs12951053 (median OR_per allele_  = 1.65; 95% CI = 1.20–2.26) in *TP53*, rs411697 (median OR _rare homozygote_  = 0.53; 95% CI  = 0.35 – 0.79) in *BACH1* and rs10131 (*median OR_ rare homozygote_  = * not estimable) in *LIG4*. The six most highly associated SNPs are either predicted to be functionally significant or are in LD with such a variant. The variants in TP53 were confirmed to be associated in a large follow-up study.

**Conclusions/Significance:**

Based on our findings, further follow-up of the DNA repair and response pathways in a larger dataset is warranted to confirm these results.

## Introduction

Ovarian cancer is the leading cause of mortality among gynecologic cancers [1]. The highly lethal serous histological type comprises about two-thirds of cases and causes most disease-related deaths. Reproductive factors such as high parity, oral contraceptive use, breast feeding, hysterectomy, and tubal ligation protect against ovarian cancer [2], whereas infertility and endometriosis increase risk [3], [4]. The biological mechanisms that underlie these risk factors are not well understood, but inflammation-related oxidative stress has been proposed as a unifying theory by which these risk factors could cause genomic damage leading to the development of cancer [5], [6], [7], [8], [9]. If this theory is correct, it is plausible that the risk of ovarian cancer would be modified by common genetic variants that affect the efficacy of DNA repair genes [10], [11].

Several lines of evidence suggest that DNA repair pathways play an important role in ovarian carcinogenesis. First, all of the high penetrance ovarian cancer susceptibility genes that have been identified thus far play a role in DNA repair. In this regard, deleterious mutations in the *BRCA1* and *BRCA2* genes reduce repair of double stranded DNA breaks. In addition, the germline mutations in DNA mismatch repair genes that cause Hereditary Nonpolyposis Colon Cancer (HNPCC) syndrome also strikingly increase ovarian cancer risk [12], [13]. Second, somatic mutations in the *TP53* gene are the most commonly acquired molecular alterations described thus far in high grade serous ovarian cancers [14], [15], [16]. *TP53* is involved in maintenance of genomic integrity via several mechanisms including induction of cell cycle arrest in response to DNA damage, DNA repair and regulation of apoptosis.

The above observations led us to hypothesize that common polymorphisms in genes associated with DNA response and repair or the p53-DNA damage checkpoint might increase ovarian cancer risk. We focused on 477 tagging single nucleotide polymorphisms (SNPs) and seven additional amino acid changing SNPs in 53 genes in DNA damage response and repair pathways. We used a Bayesian model search strategy called Multi-level Inference for SNP Association (MISA) [17] to analyze these SNPs for evidence of association with ovarian cancer using data from the population-based North Carolina Ovarian Cancer Study (NCOCS).

Bayesian methods are becoming a far more common choice for analysis of genetic association studies ([18] and references therein). This can be attributed to several factors including several key advantages the Bayesian paradigm has over the frequentist paradigm and to the increasing availability of software specifically designed for Bayesian analysis of genetic association data such as the MISA package employed here. The key shortcoming to testing in the frequentist paradigm is in its failure to explicitly account for the likelihood of the association arising under the alternative hypothesis, i.e. to account for power – data that generate a small p-value under the null may also be very unlikely under the alternate hypothesis [18]. In contrast, Bayesian methods provide measures of association – Bayes factors (BFs) and posterior probabilities – that explicitly account for the likelihood of the data under the competing hypotheses. This comes at the cost of additional modeling assumptions; namely, specification of prior probabilities for each hypothesis and prior distributions over model parameters conditional on the hypotheses.

MISA [17] improves upon SNP-at-a-time (marginal) methods by modeling phenotype as a function of a multivariate genetic profile and, as a result, provides measures of association adjusted for the remaining markers. MISA employs Bayesian Model Averaging [19], [20] to account for uncertainty in the specification of the true model of association, something that stepwise logistic regression and other model selection approaches such as lasso do not do. This has important implications: methods that identify a single model may miss important SNPs due to LD structure. In addition, MISA provides summaries of the degree to which the data support an association at the level of individual variants, genes and pathways while allowing for inference regarding the genetic parameterization (log-additive, dominant or recessive) of each SNP. The prior distribution employed by MISA was carefully chosen for the multiplicity correction it induces.

## Materials and Methods

### Study subjects

Cases and controls were participants in the NCOCS, conducted in a 48-county region of North Carolina. A detailed description of the study has been published previously [2], [21]. Briefly, cases were identified through the North Carolina Central Cancer Registry using rapid case ascertainment. Eligible cases, aged 20 to 74, were diagnosed with epithelial ovarian cancer between 1999 and 2007. Histologic slides were obtained and all cases underwent standardized histopathologic review by the study pathologist (RCB) to confirm diagnosis. The response rate among eligible cases was 70%. We found little difference in demographic and clinical characteristics among cases who participated in this study compared to those who declined. Control women were identified from the same region using random digit dialing and were frequency matched to cases by age (five-year categories) and race (black or non-black). Seventy-three percent of potential controls who passed the eligibility screening agreed to be sent additional study information. Among those sent study information, the response rate was 64 percent. Although the control response rate is somewhat low, this has not affected associations with established epidemiological risk factors [2], [21]. Additionally, it is unlikely that participation would have been influenced by genotype. The protocol was approved by the Duke University Medical Center Institutional Review Board and the human subjects committees at the Central Cancer Registry and each hospital where cases were identified.

We restricted the current analyses to white, non-Hispanic invasive serous ovarian cancer cases (n = 364) and white non-Hispanic controls (n = 761) with genotype data meeting quality control requirements. Participants had blood drawn during their in-person interview by the study nurse. Germline DNA was extracted from peripheral blood lymphocytes using PureGene DNA isolation reagents, according to manufacturer's instructions (Gentra Systems, Minneapolis, MN).

### Genotyping Methods

We selected a broad group of candidate genes in the DNA repair and response pathways (Table S1) that likely interact with *BRCA1* or *BRCA2* or are involved in double strand break, mismatch repair, nucleotide excision repair, or base excision repair. We tagged these 53 candidate genes using release 19 of the International HapMap Project's (www.hapmap.org)[22] CEU founder population and the ldSelect program [23]. We tagged the region beginning 10,000 base pairs upstream and ending 10,000 base pairs downstream of each gene so as to include the coding, non-coding and regulatory regions. ldSelect identified bins of SNPs with minor allele frequency (MAF) ≥0.05 using a pair-wise linkage disequilibrium (LD) threshold of *r* ^2^≥0.8. We chose to genotype two tags in bins where all SNPs had low Illumina design scores to improve expected coverage. For purposes of analysis, we retained the tag with the more accurate genotype calls as measured by call frequency and concordance rate in the CEPH trios. Of the 671 tagging SNPs genotyped, 61 were nonsynonymous; an additional 14 non-tagging amino acid changing SNPs were also genotyped when the tag that was chosen was also nonsynonymous. All nonsynonymous SNPs that met the criteria for the Illumina Golden Gate assays were included. The samples were genotyped using an Illumina Golden Gate Assay™ at the Duke Institute for Genome Sciences and Policy (IGSP), with cases and controls randomly mixed on each of 21 plates. Six CEPH-Utah trios (Coriell Institute, Camden, N.J.) were distributed across six plates. The plates were processed in four batches by the genotyping facility. SNPs that could not be called using the Illumina BeadStudio software on more than 1% of samples within a batch were treated as missing for that batch. We used logistic regression analysis to determine if batch and DNA quality metrics were associated with case-control status.

We evaluated the accuracy of the genetic data using SNP- and subject-specific quality control analyses. First, we removed from all association analyses SNPs with one or more CEPH genotypes in disagreement with their published values, i.e. those that had an estimated error rate greater than or equal to 1/18 assuming the published genotypes are correct. Second, we utilized the X2 goodness of fit test with continuity correction 0.25 to test for departures from Hardy-Weinberg-Equilibrium (HWE) among controls [24] and among the 60 CEPH parents using their published genotypes at the loci of interest. We removed a subset of samples on the basis of an analysis of the left tail of the distribution of p-values for HWE. This subset accounted for the Illumina GenCall 50th percentile score (GC50PCT) of each sample and used the corresponding distribution estimated from the HapMap CEPH samples for comparison. Reported estimates of minor allele frequency (MAF) are the minimum of the observed allele frequencies among controls.

Twenty-two of the 685 DNA repair SNPs on the assay had call rates below 99% on all four batches and were removed from further consideration. Thirty-seven of the remaining 663 SNPs had less than 95% concordance in the CEPH samples between our genotype calls and those published by HapMap and were removed from further consideration. Of those remaining, 484 were non-redundant and included in all subsequent analyses. A QQ plot of the HWE p-value distribution over these SNPs using all 787 white non-Hispanic controls showed evidence of an overabundance of small p-values relative to what is expected under the uniform distribution. The corresponding plot based on the HapMap genotypes of the 60 CEPH parents did not have this property.

The number of SNPs with a HWE p-value less than 0.01 calculated using all 787 white non-Hispanic controls was 17; using the HapMap sample, it was 5. Assuming the p-values are independent and uniformly distributed the expected number less than 0.01 is 4.84, the 2.5^th^ percentile of this distribution is 1 and the 97.5^th^ percentile is 10.

This suggests that, rather than having a population genetic explanation, this departure is likely due to genotyping errors. To verify this, we considered removing samples with an Illumina GC50PCT less than a threshold larger than the customary 0.7. We systematically increased the threshold up to 0.8 and found that the distribution of HWE p-values was dramatically improved at a threshold of 0.789. This choice left 364 (of 390) cases and 761 controls. Using this threshold, there were 9 SNPs with a HWE p-value less than 0.01. All further analyses were conducted using these samples and their genotype data on the 484 non-redundant SNPs passing our quality control analysis.

### Statistical Methods

#### MISA Analysis

We used MISA to identify likely associations and the genetic parameterizations of associated SNPs. MISA implements a model search over logistic regression models for case-control status given the SNP variables and a set of design and potential confounding variables. In the current analyses, age at diagnosis/interview, batch, the DNA quality metric GC50PCT, and interaction terms between batch and GC50PCT are the ‘design’ variables included in all models. We refer to the model with only the design variables as the model of ‘no genetic association,’ or ‘null’ model for short. It is
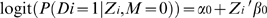
where D_i_ is the indicator of whether subject *i* is a case, M is a model identifier, α_0_ is the intercept, Z_i_ is the vector of design variables for subject *i*, and β_0_ is the vector of coefficients of the variables in Z_i_ under the null model. Adding main effects for any combination of the SNPs to the null model will define a model of association. MISA allows each included SNP to have a log-additive, dominant or recessive parameterization. MISA uses an evolutionary Monte Carlo algorithm to sample models in this class according to their posterior probabilities. This stochastic search is carried out in lieu of an enumeration of the models on account of their huge number.

Because of the astronomical number of statistical models of the above form, MISA incorporates a permissive single SNP-at-a-time (marginal) Bayes Factor (BF) screen using the entire set of non-redundant SNPs to eliminate SNPs unlikely to be associated in the multivariate logistic regression model. Wilson et al. [17] show that the screen followed by the multivariate adjusted analysis from MISA provides increased power to detect associations over the marginal analysis alone, with minimal increase in false positive rates. They show that MISA also has much better power than standard multiple comparison adjustment methods and false discovery rate procedures, stepwise logistic regression or the lasso.

MISA utilizes a prior distribution over model parameters calibrated for small to modest effect sizes and a beta-binomial prior distribution on the number of SNPs included in a model. The latter distribution induces a multiplicity correction by specifying a global prior odds of association that is independent of the number of SNPs or genes in the analysis. Its parameters, *a  = 1/8* and *b = S* (the number of SNPs in the model search phase), were chosen on basis of the results of a simulation experiment to achieve a desired balance between false positive and false negative rates. More detail on the statistical methods employed in this analysis can be found in Wilson et al. [17] (Text S1).

#### Bayesian Inference

Both the marginal and multivariate analyses use Bayes factors (BFs) to measure evidence in favor (or against) an association. The BF is equivalently a generalized likelihood ratio and an odds ratio. In the former characterization it is the ratio of the likelihood of the data under one model (e.g. a model of genetic association) to another (e.g. a model of no genetic association). Instead of taking the ratio of sampling models under each hypothesis evaluated at the most likely parameter value (MLE) of each as in the Frequentist paradigm, the BF is the ratio of the sampling models averaged over their respective prior distributions on the model parameters. In the latter characterization, BFs are defined as the ratio of the posterior odds of a hypothesis (or model) of association to the prior odds (π/(1- π)) of that hypothesis and, hence, measure the degree to which the data *update* the odds of that hypothesis of association [25], [26], [27]; with a BF of 10, the posterior odds of an association are 10 times larger than the prior odds. Under a commonly used scale of evidence [28], BFs between 1.0 and 3.2 are ‘weakly supportive’, those between 3.2 and 10 are ‘supportive’, those between 10 and 30 are ‘strongly supportive’, those between 30 and 100 are ‘very strong’ and those above 100 are ‘decisive’ for support of association (we have changed the names of several of these categories, but not their interpretation). A BF for no association is simply the reciprocal of the BF for an association, thus unlike p-values BFs can provide a measure of support in favor of a null hypothesis. BFs may be converted to posterior odds (PO  =  BF x π/(1- π)), and to posterior probabilities of association (PPA  =  PO/(1+ PO)) to provide an “absolute” measure of evidence of association[18]. The posterior probabilities may be used as part of a decision analysis to determine which SNPs to pursue further. A threshold of 0.5 for the PPA, assumes that false positives have the same cost as false negatives. For preliminary studies, a lower threshold may be more appropriate.

#### Missing Data

There were no missing design variables. We used fastPHASE [26] to generate 100 imputations of the missing genotype data given the observed, unphased genotype data. The screen's marginal BFs were calculated as the simple average of the BFs for each of the 100 imputed data sets. We compared these BFs to those calculated with a single data set in which the missing genotypes were replaced by their modal value determined from the 100 imputations. The two sets of BFs had correlation 0.998. For this reason and because computations are greatly streamlined, we used the single data set with ‘modal fill-ins’ for the MISA analysis.

Our imputation procedure assumes that missing SNP genotypes are ignorable, i.e. either missing completely at random (MCAR) or missing at random (MAR). We used the marginal BF software to check this assumption by investigating whether a SNP's pattern of missingness was conditionally independent of case-control status given the observed data we have for explaining missingness. The design variables in this analysis were the same as used in the screen and in MISA. For purposes of this calculation, we used the 0–1 indicator for a SNPs missingness in place of its genotype data and calculated BFs for association of this indicator to case-control status under the log-additive model for SNPs with five or more missing values (60 SNPs).

#### Design Variables

Logistic regression analysis of case-control status on batch and GC50PCT indicated a strong batch effect (p<10e^−7^), largely driven by an uneven allocation of cases and controls in batch four and a batch-GC50PCT interaction (p = 0.02). On the basis of this analysis, we include batch, GC50PCT, the interaction between batch and GC50PCT in all association models along with age.

#### Haplotype Analysis

Associations with one or more SNPs in a gene may occur when those variants tag a risk haplotype. We used Haploview 4.1 to carry out haplotype association tests to ascertain whether this might be the case in the genes containing the most highly associated SNPs.

## Results

### NCOCS Candidate DNA Repair Gene Analysis

In the marginal SNP-at-a-time analysis of the 484 non-redundant SNPs passing quality control, S = 77 SNPs passed the marginal screen (maximum marginal BF >1.0). (The results of the screening phase of analysis, including median odd ratios (ORs), 95% credible intervals (CIs) and MAFs for all 484 SNPs are shown in Table S2.) We ran MISA using the 77 SNPs that passed the screen with parameters *a = 1/8* and *S = 77*, which leads to marginal prior odds of association in this subset of 1/axS  = 0.0016. Table 1 lists the SNP-specific BFs for the 41 SNPs in 25 genes that had a MISA BF >1.0. The table also reports the most likely genetic model for each SNP, the posterior probability of that model and median ORs and 95% CI estimates.

**Table 1:** Results from MISA for 41 of 77 analyzed SNPs with a Bayes Factor (BFs) >1.0.

		Most Likely Model			
SNP	Gene	Model	Probability	Median OR (95% CI)*	BF
**BFs are strongly supportive of an association**					
rs5762746	CHEK2	Log Additive	0.643	0.66 (0.44, 1.00)	28.940
rs6005835	CHEK2	Log Additive	0.752	0.69 (0.53, 0.91)	28.028
rs2078486	TP53	Log Additive	0.653	1.65 (1.21, 2.25)	19.604
rs411697	BACH1	Recessive	0.988	0.53 (0.35, 0.79)	15.909
rs12951053	TP53	Log Additive	0.618	1.65 (1.20. 2.26)	14.062
rs10131	LIG4	Recessive	0.988	NA†	10.864
**BFs are supportive of an association**					
rs2287497	TP53	Log Additive	0.623	1.50 (1.14, 1.96)	9.086
rs11571424	RAD52	Recessive	0.962	7.55 (1.70, 33.47)	8.388
rs3732191	MSH6	Recessive	0.428	NA	7.589
rs16855489	XRCC5	Dominant	0.963	0.69 (0.54, 0.88)	7.353
rs32989	MSH3	Dominant	0.675	0.73 (0.57, 0.93)	6.527
rs6470522	NBS1	Recessive	0.48	1.91 (0.77, 4.75)	5.712
rs245346	MSH3	Log Additive	0.608	0.76 (0.61, 0.95)	5.493
rs929461	GADD45B	Recessive	0.932	NA	4.933
rs7307680	RAD52	Dominant	0.804	0.72 (0.55, 0.94)	4.796
rs4703819	MSH3	Recessive	0.963	NA	4.422
rs1805794‡	NBS1	Recessive	0.471	0.67 (0.42, 1.07)	3.823
rs1063045‡	NBS1	Recessive	0.557	0.66 (0.41, 1.07)	3.682
rs11571461	RAD52	Recessive	0.875	NA	3.484
rs1061302‡	NBS1	Recessive	0.55	0.66 (0.40, 1.07)	3.388
**BFs are weakly supportive of an association**					
rs1981929	MSH2	Dominant	0.900	1.40 (1.09, 1.80)	3.116
rs7546055	GADD45A	Dominant	0.709	1.32 (1.04, 1.68)	2.687
rs2832283	BACH1	Log Additive	0.586	0.70 (0.42, 1.17)	2.468
rs6151640	MSH3	Recessive	0.950	0.14 (0.02, 0.97)	2.381
rs4150383	ERCC5	Dominant	0.710	0.74 (0.58, 0.96)	2.289
rs175057	MLH3	Dominant	0.849	1.41 (1.07, 1.87)	2.169
rs2299612	FANCG	Dominant	0.847	1.37 (1.07, 1.74)	2.046
rs1011980	XRCC4	Recessive	0.938	0.48 (0.27, 0.86)	2.000
rs1498313	MSH4	Dominant	0.766	1.33 (1.02, 1.73)	1.952
rs7735781	XRCC4	Recessive	0.906	NA	1.893
rs3093933	PARP2	Recessive	0.667	0.63 (0.36, 1.11)	1.826
rs7190823‡	FANCA	Recessive	0.875	1.48 (1.09, 2.00)	1.699
rs3780560	FANCC	Recessive	0.784	NA	1.588
rs1233276	PMS1	Recessive	0.693	1.83 (1.08, 3.10)	1.460
rs2678681	PARP2	Recessive	0.799	NA	1.456
rs13292454	FANCC	Log Additive	0.463	2.11 (0.58, 7.68)	1.418
rs709816‡	NBS1	Recessive	0.723	0.65 (0.44, 0.95)	1.360
rs4253211‡	ERCC6	Log Additive	0.406	0.45 (0.11, 1.88)	1.326
rs1006548	FANCA	Recessive	0.918	1.91 (1.12, 3.25)	1.239
rs5030783	RAD51	Recessive	0.893	0.64 (0.43, 0.95)	1.236
rs769412‡	MDM2	Log Additive	0.438	1.40 (1.00, 1.96)	1.073

Abbreviations: MISA, Multilevel Inference for SNP Association Studies; SNP, single nucleotide polymorphism; BF, Bayes factor; OR, odds ratio; CI, credible interval.

* The OR corresponds to the posterior mode (equivalent to the maximum likelihood estimate (MLE)) under the normal prior distribution with mean equal to the MLE on coefficients implied by AIC under the most likely genetic model identified by MISA. The 95% CI is the 95% equal tailed posterior credible interval under the normal prior.

† The maximum likelihood estimate does not exist.

‡ Indicates the SNP is a nonsynonymous coding SNP (i.e., amino acid changing).

Of these 41 SNPs, six SNPs in four genes (*CHEK2*, *TP53*, *BACH1* and *LIG4*) have MISA BF >10 providing evidence for an association between these SNPs and ovarian cancer. These are rs5762746 (BF = 28.940) and rs6005835 (BF = 28.028) in *CHEK2*, rs2078486 (BF = 19.604) and rs12951053 (BF = 14.062) in *TP53*, rs411697 (BF = 15.909) in *BACH1* and rs10131 (BF = 10.864) in *LIG4*. Fourteen SNPs in seven genes including *GADD45B*, *MSH3*, *MSH6*, *NBS1*, *RAD52*, *TP53*, *and XRCC5* had BFs ranging from 3.39–9.09, with posterior odds that are 3.39 to 9.09 times larger than the prior odds. The SNP-specific Bayes Factors are composite measures that average over statistical models of association that include that SNP while adjusting for other potentially associated SNPs and their genetic parameterizations. Hence, they explicitly account for uncertainty in the specification of the statistical model of association.

**Figure 1:**
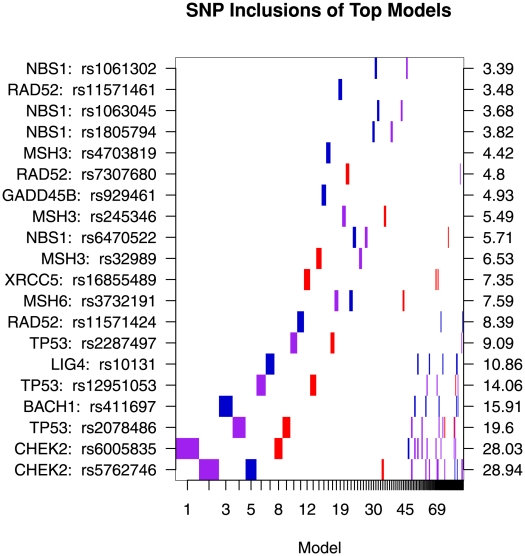
Models are ordered on the x-axis in descending posterior probability with the width of the column associated with a model proportional to the model's posterior probability. Individual SNPs are represented on the y-axis with labels giving the gene and RS number for the SNP and are ordered on the basis of the Bayes Factor in favor of SNP association, which are given on the y-axis on the right side of the plot. The presence of a SNP in a model is indicated by a colored block at the intersection of the model's column and the SNP's row, while the color of the block indicates the parameterization of the SNP: purple for log-additive, blue for recessive and red for dominant.

Figure 1 summarizes the associations of the 20 SNPs with MISA BF>3.2. This plot summarizes the top 100 models selected on the basis of their posterior model probabilities. Models are ordered on the x-axis in descending probability and the width of the column associated with a model is proportional to that probability. SNPs are represented on the y-axis. Presence of a SNP in a model is indicated by a colored block at the intersection of the model's column and the SNP's row. The color of the block indicates the genetic parameterization of the SNP in the given model: purple for log-additive, blue for recessive and red for dominant. A checkerboard pattern as opposed to a pattern of strong vertical bands indicates substantial model uncertainty. Seventy-eight of the top 100 models depicted in Figure 1, including the top 48 models, include only a single SNP in addition to the design variables. Only 22 of the top 100 models included two SNPs and none of them included more than two. The top model includes only the log-additive genetic parameterization of rs6005835 in *CHEK2* with a Maximum A Priori (MAP) estimate of the OR of 0.70. The second ranked sampled model is comprised of the log-additive genetic parameterization of rs5762746 in *CHEK2* with a MAP OR of 0.73. SNPs rs6005835 and rs5762746 in *CHEK2* have a modest LD, measured as r^2^ of 0.5.

Models that incorporate multiple SNPs represent 22 of the top 100 models by number, but only 7.8% of their total posterior mass. The low relative weighting of this class of models is largely a result of the strong MISA multiplicity correction that controls the false positive rate associated with calls made on the basis of SNP-specific BFs. The highest ranked multi-SNP model (rank = 49) includes CHEK2 rs5762746 and TP53 rs2078486. These variants are complementary predictors, each explaining as much variability in case-control status when modeled alone as when modeled in the presence of the other. This suggests that SNPs from multiple genes related to DNA damage repair provide complementary information for characterizing ovarian cancer risk.

#### Haplotype Analysis

A Haploview [29] analysis of CHEK2, TP53, BACH1 and LIG4, containing the top six SNPs, showed no evidence for multi-SNP (haplotype-based) risk genotypes.

#### Analysis of missing data

Of the 60 SNPs with more than 4 missing SNP genotypes, only six had BFs>1.0 for conditional dependence of case-control status on missingness given the design variables. Four of these BFs were in the range from 1.01 to 1.49 and are not of concern. The remaining two, rs11571789 in BRCA2 (BF = 3.80) and rs1805794 in NBS1 (BF = 4.60), were ‘supportive’ of association. These may be due either to chance or to the presence of an unmeasured confounder and reflect a pattern of non-ignorable missingness. Missing data imputations that ignore the possibility of low frequency polymorphisms which interfere with the ability to assay a probe will not account for the LD between the rare associated variant and the SNP with missing values. The ultimate effect when fitting the association models for this SNP will be for the SNPs apparent effect to be biased. BRCA2 rs11571789's maximum marginal BF for association with ovarian cancer was 0.26 (‘supportive’ of no association) and was not included in the MISA analysis. NBS1 rs1805794's maximum marginal BF was 1.76 and its MISA BF was 3.82. This modest evidence in favor of association should be interpreted in light of the potential for this effect to have been confounded.

## Discussion

The results of this study provide evidence for an association between several genes in the DNA repair and response pathways and risk of invasive serous ovarian cancer. There was strong support for associations between ovarian cancer and two SNPs in *CHEK2*, two SNPs in *TP53*, *and* one SNP each in *BACH1*, *and LIG4*. Our analyses are also supportive of associations between four SNPs in *NBS1*, three SNPs in *MSH3*, three SNPs in *RAD52*, and one SNP each in *GADD45B*, *MSH6*, *TP53*, *and XRCC5* and invasive serous ovarian cancer. To our knowledge, this is the first study suggesting associations between ovarian cancer and SNPs in *CHEK2*, *BACH1*, *XRCC5*, *NBS1*, *MSH6*, *RAD52*, *and GADD45B*. As discussed below, there is evidence that several of the most highly associated SNPs may have functional significance.

We used SNPInfo analysis [30] to determine if any of the six SNPs with a MISA BF >10 were in LD with a putative functional variant or are predicted to have functional significance. We examined each HapMap SNP with LD of 0.5 or higher to one of the six top SNPs. Table S3 reports whether the variant is predicted to affect a transcription factor binding site, a splicing site, an miRNA binding site or alter the structure of a protein product. In addition, it indicates whether the SNP is a non-synonymous or nonsense variant and reports its regulatory potential and sequence conservation scores. Based on this analysis, both rs10131 in *LIG4* and rs9587535 in ABHD13, a SNP in high LD with rs10131 (LD = 0822) are predicted by miRanda [31] to affect an miRNA binding site. In addition, rs10131 has a high predicted sequence conservation score (for a non-coding variant). Two other *LIG4* LD SNPs (rs1931336 and rs9587535 with LD 0.59 and 0.82 with rs10131, respectively) also have this property. Several variants in weak LD (0.5<LD<0.7) with *TP53* rs12951053 are predicted to affect a transcription factor binding site; one of these (rs17882227) is in perfect LD with *TP53* rs2078486, one of the most highly associated SNPs. In addition, rs2287498 in *WDR79* (in perfect LD with rs2078486 and in LD (R^2^ = 0.62) with rs12951053) is predicted to affect function at a splice site and a non-synonymous variant (rs2287499) in *WDR79* in weak LD with the two most highly associated *TP53* SNPs is predicted by PolyPhen [32] to be benign. Several of the non-coding *TP53* variants have high regulatory potential and/or sequence conservation scores; of these rs17882227 is in highest LD (1.0) with a top candidate (rs2078486). SNP rs388707 in LD with *BACH1* rs411697 is predicted to affect splicing, while another SNP (rs425989) in LD with rs411697 is predicted by miRanda to affect an miRNA binding site. In addition, three intronic SNPs in LD with our *BACH1* candidate have sequence conservation scores greater than 0.1, suggesting that they may be functionally interesting. Several variants in and near *CHEK2* demonstrate the potential to affect function. There are a number of non-coding *CHEK2* SNPs (e.g. rs5762629, rs2346397 and the top SNP rs6005835) with high regulatory potential or sequence conservation scores. In addition to having a high regulatory potential score, SNP rs6005835 in *CHEK2* is predicted to affect a transcription factor binding site.

In addition to the functional significance, a recently published multi-center study [33] (which included data from the NCOCS) examined six SNPs in TP53 and validated our findings (with and without the NCOCS data). SNPs with strong evidence for association in our study include two tagging SNPs in *TP53*, rs12951053 and rs2287497, both of which were replicated with posterior median ORs of 1.19 (95% CI: 1.01, 1.38) and 1.30 (95% CI: 1.07, 1.58), respectively [33]. A third SNP in *TP53*, rs2078486, was also associated with ovarian cancer in three independent datasets in the same report. Although our data support that two SNPs in the CHEK2 gene are associated with the risk of developing invasive serous ovarian cancer, it is of note that one prior study in Poland did not find evidence for an association between invasive epithelial ovarian cancer of any histologic subtype and three founder alleles in the CHEK2 gene but did find evidence for an association with ovarian tumors of low malignant potential and the CHEK2 I157T missense variant {Szymanska-Pasternak, 2006 #447}.

Other studies that have assessed associations between variants in the DNA repair genes included in the current analysis and ovarian cancer risk [34], [35], [36], [37] are summarized in Table 2. Differences in the findings between studies when the SNPs were in high LD may be explained by differences in the analytic approach and the mode of inheritance analyzed. Our approach included a two-staged analysis plan that provided increased power to detect SNP associations and accounted for multiple modes of inheritance. A strength of the current study was the restriction to invasive serous ovarian cancers, whereas previous reports often combined histologic subtypes. By restricting to the serous invasive subtype, we achieve a more homogenous subset of ovarian cancers likely to have similar etiology and genetic factors and avoid diluting the magnitude of associations. In addition, inclusion of prevalent, rather than only incident, cases may be a source of inconsistency between studies.

**Table 2:** Comparison of SNPs with MISA Bayes Factors >1.0 to previously published data.*

Gene	Associated SNP(s) from NCOCS	MISA BF for associated SNP(s) from NCOCS	SNPs examined in previous studies	Maximum r^2^ and/or r^2^≥0.75 between associated NCOCS SNP and SNP from previous study
***TP53***	**rs2078486 rs12951053 rs2287497**	19.60414.0629.086	**rs2078486** [33] † **rs12951053** [33] † **rs2287497** [33] †	1.00 (**rs2078486, rs2078486**)1.00 (**rs12951053, rs12951053**)1.00 (**rs2287497, rs2287497**)
***NBS1***	rs6470522 **rs1805794 rs1063045 rs1061302 rs709816**	5.7123.8233.6823.3881.360	**rs1805794** [34] **rs1063045** [34] **rs1061302** [34] **rs709816** [34]	1.00 (**rs1805794, rs1805794**)1.00 (**rs1061302, rs1061302**)1.00 (**rs1063045, rs1063045**)1.00 (**rs709816, rs709816**)
***LIG4***	rs10131	10.864	rs1805386[37]	0.02 (rs10131, rs1805386)
***MLH3***	rs175057	2.169	rs7303[35]rs175080[35]	0.93 (rs175057, rs175080)0.78 (rs175057, rs7303)
***MSH2***	rs1981929	3.116	rs4952887[35] ‡rs2303428[35] ‡rs1981928[35]rs2059520[35]rs3771274[35]rs3771281[35]rs13425206[35]	0.44 (rs1981929, rs3771274)
***MSH3***	rs32989rs245346rs4703819rs6151640	6.5275.4934.4222.831	rs26279[35]rs10079641[35]rs6151662[35] †rs26282[35]rs26779[35]rs33008[35]rs40139[35]rs184967[35]rs2112416[35]rs2897298[35]	1.00 (rs32989, rs26279)1.00 (rs6151640, rs10079641)
***MSH6***	rs3732191	7.589	rs2348244[35]rs3136245[35] †rs1800932[35]rs1800935[35]rs3136272[35]rs3136317[35]rs2020911[35]	0.40 (rs3732191, rs2348244)
***PMS1***	rs1233276	1.460	rs3762545[35]rs5742981[35]rs5741593[35]rs1233291[35]rs1233255[35]rs1233258[35]rs256571[35]rs256563[35]	0.85 (rs233275, rs1233255)
***RAD52***	rs11571424rs7307680rs11571461	8.3884.7963.484	rs11226[34]rs4987208[38]	0.06 (rs11571461, rs11226)
***RAD51***	rs5030783	1.236	rs1801320[34]rs1801321[34]	0.48 (rs5030783, rs1801321)

Abbreviations: NCOCS, North Carolina Ovarian Cancer Study; SNP, single nucleotide polymorphism; BF, Bayes factor; MISA, Multilevel Inference for SNP Associations.

NOTE: SNPs in bold font are common to both the current NCOCS study and to previously reported studies.

* Previous reports include: Multiple reports from the same study;[34], [35], [36] one report that used data from two related case-control studies in Australia;[38] one Ovarian Cancer Association Consortium (OCAC) meta-analysis[37] that includes overlap of data from the Auranen et al.[34] and Beesley et al.[38] studies; and one OCAC analysis that includes data from the current NCOCS study[33].

† SNP was statistically significantly associated with ovarian cancer in the previous study.

‡ The ORs for the heterozygote genotypes, but not the homozygote rare genotypes, were statistically significant. The overall p- trend values were greater than 0.05.

§ SNP was statistically significantly associated with ovarian cancer in the Auranen et al.[34] study but not the other two published studies.

Despite the relatively small sample size (364 cases/761 controls) our analyses provide supportive evidence for associations with several candidate genes in the DNA repair and response pathways. Additionally, we have found evidence to suggest that the six most highly associated SNPs either may have functional significance or are in LD with a functional variant. Future work to replicate and characterize these associations in serous ovarian cancer is needed, as well as an examination of the three other important histologic subtypes of invasive ovarian cancer including mucinous, endometrioid and clear cell cancers. Because ovarian cancer is a leading cause of gynecologic cancer morbidity and mortality and DNA repair and response is a vitally-important pathway, the identification of genes and genetic variants in these pathways using a well-informed selection of SNPs may lead to the identification of genes for targeted preventive studies.

## Supporting Information

Table S1 Description of the 53 genes related to DNA Repair and p53-mediated damage response pathways.
(0.02 MB XLS)

Table S2 Marginal screen results for 484 DNA Repair SNPs in the North Carolina Ovarian Cancer Study.
(0.12 MB XLS)

Table S3 Functional annotation data for the six SNPs with a MISA Bayes Factor >10.
(0.05 MB XLS)

Text S1 Expanded description of the statistical analytic approach.
(0.12 MB PDF)
