# Supplementary material for: Association between DNA Damage Response and Repair Genes and Risk of Invasive Serous Ovarian Cancer
Source: PLoS One. 2010 Apr 8;5(4):e10061. doi: 10.1371/journal.pone.0010061 (PMC2851649; doi:10.1371/journal.pone.0010061)
Supplement: Text S1 — Expanded description of the statistical analytic approach. (0.12 MB PDF) [file pone.0010061.s004.pdf]

SUPPLEMENTAL MATERIAL MISA: ASSOCIATION BETWEEN 53 DNA DAMAGE RESPONSE AND REPAIR GENES AND RISK OF INVASIVE SEROUS OVARIAN CANCER

MISA is a methodology developed for candidate gene/pathway association studies involving single nucleotide polymorphisms (SNPs) that addresses two questions: "To what extent does the data support an overall association between a set of SNPs within a pathway and outcome of interest?" and "Which genes/SNPs are most likely driving the association?". Using a logistic regression framework. MISA improves on the more common single SNP-at-a-time methods by modeling the outcome variable as a function of a multivariate genetic profile, which provides measures of association at the global (pathway), gene, and SNP levels that are adjusted for the remaining genetic markers. In particular, we use logistic regression models to relate disease status to a set of design or confounding variables (such as age, race, previous diagnosis of cancer etc.), henceforth called design variables, and subsets of SNPs, where we consider both the choice of SNPs as well as the genetic parametrizations for each SNP that may be included in a model. An individual model, denoted by $\mathcal{M}_{\boldsymbol{\gamma}}$, is specified by the S dimensional vector γ, where S is the total number of SNPs under study and γ_s indicates the inclusion and SNP-specific genetic parametrization of SNPs in model $\mathcal{M}_{\boldsymbol{\gamma}}$. Thus for each individual, the logistic regression under model $\mathcal{M}_{\boldsymbol{\gamma}}$ is given by

$$\text{logit}(p(D_i = 1|\mathbf{z}_i, \mathbf{x}_{\boldsymbol{\gamma}_i}, \boldsymbol{\theta}_{\boldsymbol{\gamma}}, \mathcal{M}_{\boldsymbol{\gamma}})) = \alpha_0 + \mathbf{z}_i^T \boldsymbol{\alpha} + \mathbf{x}_{i\boldsymbol{\gamma}}^T \boldsymbol{\beta}_{\boldsymbol{\gamma}}$$

where $\mathbf{z}_i$ is the vector of design/confounding variables that are included in all models, $\mathbf{x}_{i\boldsymbol{\gamma}}$ represents the coding of SNPs included in model $\mathcal{M}_{\boldsymbol{\gamma}}$ and $\boldsymbol{\theta}_{\boldsymbol{\gamma}}$ is the vector of model specific parameters $(\alpha_0, \boldsymbol{\alpha}^T, {\boldsymbol{\beta}_{\boldsymbol{\gamma}}}^T)$, with intercept α_0, vector of design variable coefficients $\boldsymbol{\alpha}$, and log–odds ratios $\boldsymbol{\beta}_{\boldsymbol{\gamma}}$ for the included SNPs.

MISA uses Bayesian Model Averaging (BMA) (see Hoeting *et al.* [1999] or Clyde and George [2004] for overviews) to combine information from multiple models, $\mathcal{M}_{\boldsymbol{\gamma}}$, to address the degree to which the data support an association at the level of individual SNPs, genes and pathways, while taking into account uncertainty regarding the best genetic parametrizations. By using model averaging, MISA improves upon methods that select a single model and is effective in identifying subsets of likely associated variables, for prioritizing them and for measuring overall association in the presence of

model uncertainty. In BMA, posterior probabilities of the individual models, $\mathcal{M}_{\boldsymbol{\gamma}}$ can be calculated once prior probabilities of each model are specified, $p(\mathcal{M}_{\boldsymbol{\gamma}})$. Then, Bayes Factors (BF) [Kass and Raftery 1995], which compare the posterior odds of any two models (or hypotheses) to their prior odds, are used to measure the change in evidence provided by the data for one model, $\mathcal{M}_{\boldsymbol{\gamma}_1}$, to another, $\mathcal{M}_{\boldsymbol{\gamma}_2}$ or for pairs of hypothesis. In particular in MISA, BFs are defined for quantifying associations at multiple levels (global, gene, and SNP).

As mentioned above, to calculate the multi-level Bayes Factors of interest one must specify the prior probability of each model $\mathcal{M}_{\boldsymbol{\gamma}}$. In MISA, these priors are specified based on the prior inclusion probability of any given SNP within a model. A priori the SNPs are taken to be *exchangeable*, that is the prior distribution of the indicator variables $\boldsymbol{\gamma}$ does not depend on the order of the SNPs. This assumption may be represented by a hierarchical model where the inclusion of any SNP in the model is assumed to be and independent and identically distributed from a Bernoulli distribution, with inclusion probability θ for any SNP, and assigning a prior distribution to θ. This leads to a prior distribution on the model size $s_{\boldsymbol{\gamma}}$ that is Binomial, $\mathsf{Bin}(S, \theta)$. We place a $\mathsf{Beta}(a, b)$ hyper-prior on the quantity θ, which leads to the $\mathsf{BetaBinomial}(a, b)$ distribution on the number of included SNPs. This distribution provides over-dispersion, added robustness to prior misspecification, and an implicit multiplicity correction as a function of the total number of SNPs under study compared to the $\mathsf{Bin}(S, \theta)$ distribution. We recommend $a = 1$ as a default, so that the prior distribution on model size is non-increasing in $s_{\boldsymbol{\gamma}}$ (a geometric distribution). The hyper–parameter b can then be chosen to reflect the expected model size, global prior probability of at least one association, or the marginal prior odds that any SNP is associated.

The choices for hyperparameters have implications for the global Bayes factor. The $\mathsf{BetaBinomial}(1, 1)$ has a global prior odds of association equal to the number of SNPs, S, and would be appropriate for the case where increasing the number of SNPs under consideration reflects increased prior certainty that an overall (global) association should be detected. Under the $\mathsf{BetaBinomial}(1, \lambda S)$, the global prior odds are constant, $1/\lambda$, reflecting a prior odds for overall association that is independent of the number of genes/SNPs tagged. The $\mathsf{BetaBinomial}(1, \lambda S)$ provides an implicit multiple testing penalty in the number of SNPs (rather than tests) that are included in the study of interest by keeping the global (pathway) prior odds of an association constant while decreasing the marginal prior odds of any one of the SNPs being associated as the number of SNPs increases. As a skeptical

"default" prior, we suggest the hyper–parameters $a = 1$ and $b = S$ which leads to the global prior odds of there being at least one association of 1 and the marginal prior odds of any single SNP being associated of $1/S$.

Given the number of SNPs under consideration, enumeration of all models for S greater than 25–30 is intractable. While it is possible to enumerate all single variable SNP models, the number of models with 2 or 3 SNPs allowing for multiple genetic parametrizations is in the millions or more for a typical modern hypothesis–oriented study. Stochastic variable selection algorithms provide a more robust search procedure than stepwise methods, but also permit calculation of posterior probabilities and Bayes factors based on a sampling a set of the most likely candidate models from the posterior distribution. MISA makes use of a stochastic search algorithm that is based on Evolutionary Monte Carlo (EMC) [Liang and Wong 2000], which is a mixture of parallel tempering and a genetic search algorithm. Efficiency of stochastic algorithms often diminishes as the total number of models increases. For this reason, we have found it useful to reduce the number of SNPs included in the EMC search using a screen when S is large. Such a screen will typically be fairly permissive, leaving only the weakest candidates out of the stochastic search. The screen should be quick to calculate, adjust for the same design variables and consider the same genetic parametrizations as in the full analysis. In our analyses, we calculated marginal (i.e. SNP-at-a-time) Bayes Factors for each of the log-additive, dominant and recessive models of association against the model of no association. We ordered SNPs according to the maximum of the three marginal Bayes factors and retained those with a maximum marginal BF greater than or equal to one.

We used simulated case–control data to compare MISA and other procedures. The simulated data sets were structured so as to reflect the details — genes, tag SNPs, LD structure, and sample size — of the North Carolina Ovarian Cancer Study (NCOCS) candidate pathway study comprised of 53 genes tagged by 508 tag SNPs. Using a simulation study, we have evaluated MISA against established, simple to implement and more commonly used methods, such as single (marginal) SNP-at-a-time methods using FDR corrections, stepwise logistic regression, Lasso, and logic regression, and demonstrated that our methodology outperforms these methods in detecting associations in modestly powered candidate pathway case–control studies. The improvement in power is most noticeable for odds ratios of modest (real world) magnitude and comes at the cost of only a minimal increase in the false positive rate. Like stepwise logistic regression, lasso and logic regression, MISA improves upon marginal, SNP–at–a–time methods by considering multivariate adjusted associations. By using model averag-

ing, MISA improves upon these multivariate methods that select a single model, which may miss important SNPs because of LD structure. These improvements have concrete implications for data analysis: MISA identified SNPs in the NCOCS data that were subsequently externally validated; none of the less complex methods considered here highlighted these SNPs to be of interest. At the time of preparing this supplement, other top ranked SNPs in genes identified by MISA are undergoing external validation. Finally, we note that while MISA was developed for binary outcomes in case-control studies, MISA is readily adaptable to accommodate other forms of outcome variables (e.g. quantitative traits or survival) that are naturally modeled within a GLM framework.

Web Resources. The URL for R software for the methodology presented in this supplement is:

`http://stat.duke.edu/gbye/MISA.html`

For more technical details regarding MISA, prior choices, simulation studies, and illustrations, please refer to Wilson *et al.* [2009].

References.

Clyde, M. and George, E. I. (2004). Model uncertainty. *Statist. Sci.* **19** 81–94.

Hoeting, J. A., Madigan, D., Raftery, A. E. and Volinsky, C. T. (1999). Bayesian model averaging: a tutorial (with discussion). *Statist. Sci.* **14** 382–401. Corrected version at http://www.stat.washington.edu/www/research/online/hoeting1999.pdf.

Kass, R. E. and Raftery, A. E. (1995). Bayes factors. *J. Amer. Statist. Assoc.* **90** 773–795.

Liang, F. and Wong, W. H. (2000). Evolutionary monte carol: Applications to c_p model sampling and change point problem. *Statistica Sinica* **10** 317–342.

Wilson, M. A., Iversen, E. S., Clyde, M. A. and Schildkraut, S. C. S. J. M. (2009). Bayesian model search and multilevel inference for snp association studies. Tech. rep., Available on ArXiv at http://arxiv.org/abs/0908.1144v1.
